# Bone Marrow Microenvironment-Induced Chemoprotection in *KMT2A* Rearranged Pediatric AML Is Overcome by Azacitidine–Panobinostat Combination

**DOI:** 10.3390/cancers15123112

**Published:** 2023-06-08

**Authors:** Kara M. Lehner, Anilkumar Gopalakrishnapillai, Edward Anders Kolb, Sonali P. Barwe

**Affiliations:** 1 Lisa Dean Moseley Foundation Institute for Cancer and Blood Disorders, Nemours Children’s Hospital, Wilmington, DE 19803, USA; kmlehner@udel.edu (K.M.L.); anil.g@nemours.org (A.G.); eakolb@nemours.org (E.A.K.); 2 Department of Biological Sciences, University of Delaware, Newark, DE 19716, USA

**Keywords:** acute myeloid leukemia, azacitidine, chemosensitivity, coculture, epigenetics, panobinostat, pediatric, xenograft

## Abstract

**Simple Summary:**

Leukemic cells evade chemotherapy due to interactions within the bone marrow microenvironment. We have tested the effect of epigenetic therapy on pediatric AML cells in multicell coculture and in murine models. Epigenetic drug combination followed by chemotherapy was effective in overcoming chemoprotection in multicell coculture and increasing survival in *KMT2A* rearranged murine models.

**Abstract:**

Advances in therapies of pediatric acute myeloid leukemia (AML) have been minimal in recent decades. Although 82% of patients will have an initial remission after intensive therapy, approximately 40% will relapse. *KMT2A* is the most common chromosomal translocation in AML and has a poor prognosis resulting in high relapse rates and low chemotherapy efficacy. Novel targeted approaches are needed to increase sensitivity to chemotherapy. Recent studies have shown how interactions within the bone marrow (BM) microenvironment help AML cells evade chemotherapy and contribute to relapse by promoting leukemic blast survival. This study investigates how DNA hypomethylating agent azacitidine and histone deacetylase inhibitor panobinostat synergistically overcome BM niche-induced chemoprotection modulated by stromal, endothelial, and mesenchymal stem cells and the extracellular matrix (ECM). We show that direct contact between AML cells and BM components mediates chemoprotection. We demonstrate that azacitidine and panobinostat synergistically sensitize MV4;11 cells and *KMT2A* rearranged pediatric patient-derived xenograft lines to cytarabine in multicell coculture. Treatment with the epigenetic drug combination reduced leukemic cell association with multicell monolayer and ECM in vitro and increased mobilization of leukemic cells from the BM in vivo. Finally, we show that pretreatment with the epigenetic drug combination improves the efficacy of chemotherapy in vivo.

## 1. Introduction

Acute myeloid leukemia (AML) is an aggressive hematological malignancy, resulting in a block in differentiation and the uncontrolled proliferation of mutated immature myeloblasts in the bone marrow affecting normal hematopoiesis. Chromosomal translocations involving the *KMT2A* gene have a poor prognosis and account for 35% of myeloid leukemias [1]. The high rate of relapse and drug resistance in AML is partially due to the role of the bone marrow microenvironment in leukemogenesis. In the bone marrow microenvironment, AML cells are able to closely interact with other cell types, such as endothelial, stromal and mesenchymal stem cells (MSCs), and extracellular matrix (ECM) proteins, including collagen, osteopontin, and fibronectin [2,3,4,5,6]. Cross talk between cells initiates anti-apoptotic signaling pathways that enable leukemic cells to evade chemotherapy [4,6,7].

Treatment options to overcome the effects of chemoprotection in the protective bone marrow niche are essential to decrease relapse rates in pediatric AML. The expression of cell adhesion molecules (CAMs), such as E-selectin, can be altered after epigenetic drug treatments leading to a decrease in adhesion of these molecules to the bone marrow microenvironment and increased chemotherapy treatment efficacy [8]. Aberrant DNA methylation and histone acetylation modifications are a common characteristic of leukemogenesis and modulate drug resistance. Currently, azacitidine and panobinostat are being used in clinical trials both separately and in combination in adult AML [9]. Moreover, translocations within the *KMT2A* gene are more frequent than mutations in children compared to adults [1]. As pediatric *KMT2A* rearranged leukemia is driven by fusion proteins that modify the cell’s epigenetic landscape, drugs that reverse epigenetic modifications by impacting the chromatin structure would make an excellent therapeutic target [10].

In this study, we investigate the ability of azacitidine, a DNA hypomethylating agent, and panobinostat, a histone deacetylase inhibitor, to overcome endothelial, stromal, and MSC-induced chemoprotection in pediatric AML samples with *KMT2A* rearrangement. We show that multicell coculture induced greater chemoprotection by cell–cell and cell–ECM contact. Pretreatment with an azacitidine–panobinostat combination sensitized leukemic cells in multicell coculture to traditional chemotherapy ex vivo in a variety of patient-derived xenograft (PDX) samples with differing *KMT2A* rearrangements. Treatment with the epigenetic drug combination reduced leukemic cell association with multicell monolayer and ECM in vitro and increased mobilization of leukemic cells from the bone marrow in vivo. Finally, we show that pretreatment with the epigenetic drug combination improves the efficacy of chemotherapy in vivo in MV4;11 and NTPL-146 xenografted mice.

## 2. Materials and Methods

### 2.1. Cell Culture, Patient Samples, and Reagents

MV4;11 (CRL-9591), HUVEC (CRL-1730), and HS5 (CRL-11882) cells were obtained from American Type Culture Collection (ATCC), Manassas, VA, USA. MSCs were generated in the laboratory from pediatric bone marrow as described previously [11]. MV4;11 cells were cultured in IMDM medium and supplemented with 10% fetal bovine serum (FBS), 2 mM/L L-glutamine, 25 U/mL penicillin, and 25 mg/mL streptomycin. HS5 cells were cultured in DMEM media with supplements described above. HUVEC cells were cultured in endothelial cell growth media with 2% FBS, 5 ng/mL vascular endothelial growth factor (VEGF), 15 ng/mL insulin-like growth factor (IGF), 5 ng/mL epidermal growth factor (EGF), 5 ng/mL fibroblast growth factor (FGF), and 1 μg/mL hydrocortisone hemisuccinate (HSE). MSC cells were cultured in α-MEM and supplemented with 16% FBS.

Primary AML cells isolated from bone marrow aspirates or the peripheral blood of patients treated at Nemours Children’s Hospital were collected from the Nemours Biobank under a Nemours Delaware Institutional Review Board (IRB) protocol approved by the Nemours Office of Human Subjects Protection. Patient samples CBAM-44728 and CBAM-68552 referred to as DF-5 and DF-2, respectively, were obtained from the Dana Farber Cancer Institute PRoXe depository [12]. NEM10 PDX was generated using patient samples obtained from the Children’s Oncology Group. PDX cell lines were authenticated using the ampFLSTR Identifiler PCR Amplification Kit (ThermoFisher Scientific, Waltham, MA, USA) and were confirmed by PCR analysis to be free of mycoplasma contamination. Patient samples were passaged in mice following the guidelines of the Nemours Institutional Animal Care and Use Committee (IACUC), as described previously. Mouse-passaged primary AML cells were used for ex vivo studies.

Azacitidine (S1782), panobinostat (S1030), cytarabine (S1648), and daunorubicin (S3035) were obtained from Selleckchem (Houston, TX, USA). The powder was dissolved in DMSO to appropriate concentrations for the treatments. MaxGel (E0282) was obtained from Millipore Sigma (Burlington, MA, USA). Cytokines were obtained from R&D Systems (Minneapolis, MN, USA).

### 2.2. Determination of Cell Viability in Multicell Coculture

A total of 6000 HUVEC, 1000 MSC, and 3000 HS5 cells (6:1:3 ratio to maintain optimum cell viability) were plated on 96-well plates and left to adhere for 24 h. Media was removed and cells stained with violet proliferation dye (VPD450) (30,000 MV4;11 or 60,000 cells for PDX cells) were plated in IMDM medium and indicated drugs were added to each well for 48 h. Cell viability was analyzed via flow cytometer.

### 2.3. Measuring the Effect of Soluble Factors in Chemoprotection with Conditioned Medium

To identify if soluble factors play a role in adherent cell-mediated chemoprotection, 90,000 HUVEC, 45,000 HS5, and 15,000 MSC were plated in a 6-well plate in IMDM medium and left to adhere for 48 h. Media was collected from the 6-well plate and filtered through a 0.2 μm filtered syringe and diluted to 50% with complete IMDM media. MV4;11 cells (30,000) were plated in 50% conditioned media and treated with 3 μM cytarabine for 48 h. Cell viability was analyzed via flow cytometer.

### 2.4. Adhesion Assay

Leukemic cells were cultured with or without azacitidine (aza) (1 μM) and panobinostat (pano) (1 μM) for 48 h in a 12 well plate. VPD450-stained leukemic cells (75,000), with and without epigenetic drug treatment, were plated on HUVEC, HS5, and MSC monolayers in 96-well plates for 4 h. Then, the 96-well plates were coated with 1 mg MaxGel for 1 h at 37°. Unbound cells were removed by PBS wash. After trypsinization, the number of adherent MV4;11 cells were identified by VPD staining via flow cytometry.

### 2.5. Sensitivity Assay

Leukemic cells (30,000) were plated in a 96-well plate and pre-treated with azacitidine–panobinostat or left untreated for 48 h. Cells were then transferred onto corresponding wells containing 6000 HUVEC, 3000 HS5, and 1000 MSC that were plated the previous day and left to adhere 24 h. Cells were treated with cytarabine for another 48 h. Cell viability was analyzed via flow cytometer.

For PDX lines, 60,000 leukemic cells were plated in a 96-well plate and pre-treated with indicated concentrations of azacitidine and panobinostat for 24 h and then transferred onto a monolayer of HUVEC, MSC, and HS5 cells and treated with indicated concentrations of cytarabine or daunorubicin.

### 2.6. Xenograft Studies

NSG-B2m mice (Stock#010636) purchased from Jackson Laboratories (Bar Harbor, ME, USA) were transplanted with leukemic cells (3.5 × 10^6^) by tail vein injections. Mice were maintained in the Nemours Life Science Center following the guidelines established by the Nemours IACUC. Following engraftment confirmation, mice were assigned to four different treatment groups: (1) vehicle treatment (5% dextrose), (2) epigenetic therapy consisted of azacitidine and panobinostat (daily for 5 doses, 2.5 mg/Kg each, i.p.), (3) chemotherapy consisting of cytarabine (daily for 5 doses, 50 mg/Kg, i.p.) and daunorubicin (daily for 3 doses, 1.5 mg/Kg, i.v.) as described previously [13,14], (4) epigenetic therapy was followed by chemotherapy after one day break in between. Leukemic burden was measured via flow cytometry of mouse peripheral blood drawn by submandibular bleeds [15,16]. Mice were monitored daily, and once mice reached experimental endpoints they were euthanized.

### 2.7. Statistical Analysis

All the results are expressed as means ± standard deviation (SD). All single-parameter measurement comparisons were determined using the Student’s *t*-test (Graph-Pad Prism 9 software, GraphPad, San Diego, CA, USA). Treatments were carried out in duplicates or triplicates, and in 3–4 independent experiments. All tests were two-sided; *p* of <0.05 indicates statistical significance. For group comparisons, two-way ANOVA was used.

## 3. Results

### 3.1. Multicell Coculture Exhibits Greater Chemoprotection to AML Cells

**Figure 1:**
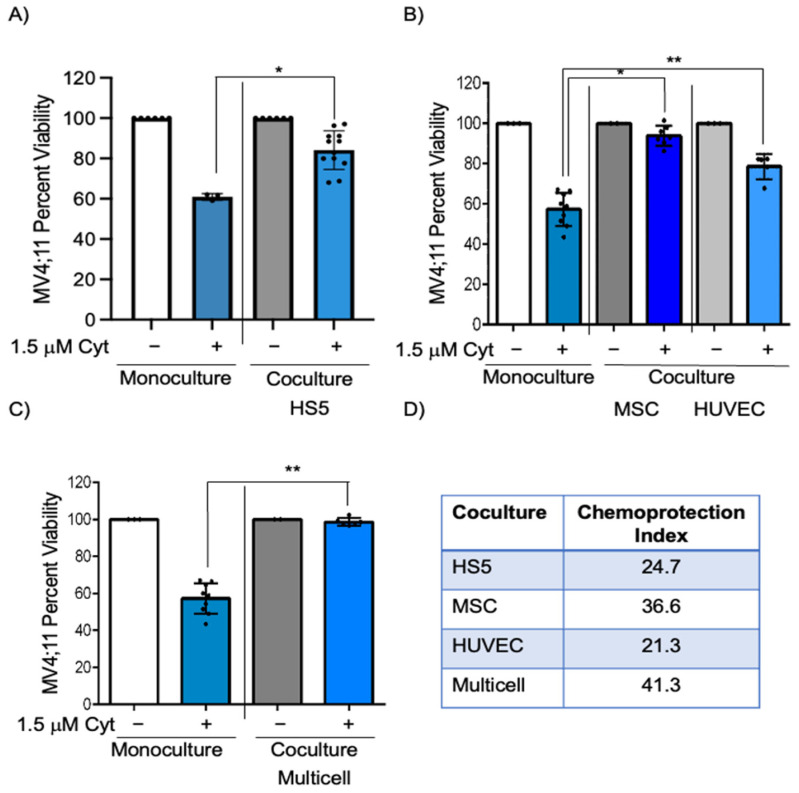
MV4;11 cells in coculture and multicell coculture showed greater viability when exposed to cytarabine. (**A**) MV4;11 cells were treated with 1.5 μM cytarabine in the presence or absence of HS5 stromal cells. (**B**) Cells were treated with 1.5 μM cytarabine with or without HUVEC endothelial cells or MSCs. (**C**) MV4;11 cells were treated with 1.5 μM cytarabine in the presence or absence of multicell coculture with, stromal, MSC, and endothelial cells in a 6:3:1 ratio. (**D**) Chemoprotection index was calculated for HS5, MSC, HUVEC, and multicell coculture. Error bars display the SD from the mean of at least three independent experiments in duplicates. Each black dot represents individual value. * *p* < 0.05, ** *p* < 0.01, level of confidence to determine statistical significance.

The bone marrow microenvironment plays a vital role in chemoprotection [2,3,7,17,18] resulting in leukemia progression. In this study, we determined if there is a reduction in cytarabine-induced killing of AML cells in coculture with HS5, a human stromal cell line isolated from bone marrow, compared to monoculture. The 1.5 μM cytarabine had decreased efficiency in inducing cell death as this treatment showed 60.9% and 85.6% viability in monoculture and coculture with HS5 cells, respectively (Figure 1A, *p* < 0.03). The chemoprotection index between coculture and monoculture (calculated by subtracting the viability percentage in monoculture from that in coculture) was 24.7, indicating significant HS5-induced protection when MV4;11 cells are exposed to cytarabine. We determined chemoprotection afforded by endothelial (HUVEC) and MSCs that also comprise the bone marrow microenvironment. The chemoprotection index in MSC coculture was 36.6 (Figure 1B bars 2 and 4, *p* < 0.05), while that for endothelial cells was 21.3 (Figure 1B, bars 2 and 6, *p* <0.01), indicating the highest chemoprotection index in MSC coculture.

**Figure 2:**
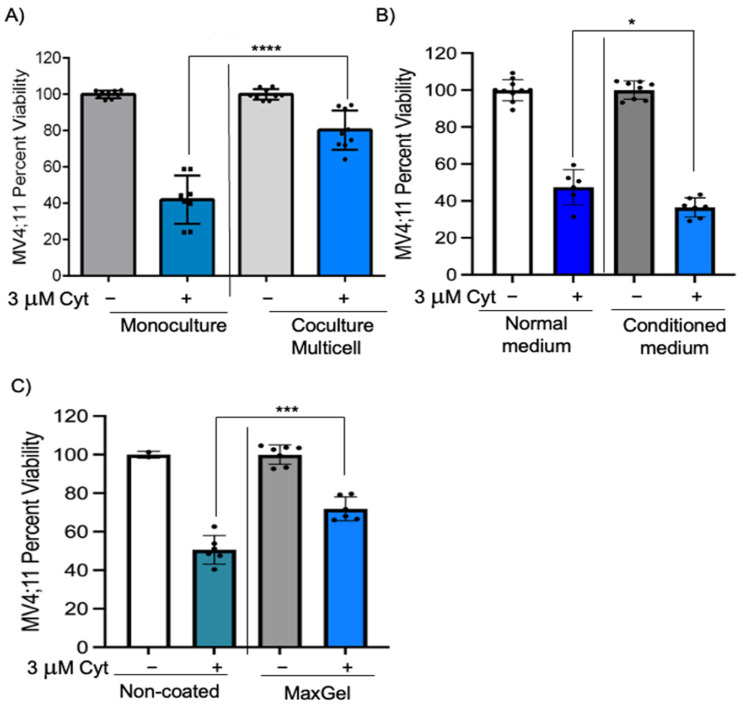
Chemoprotection is significantly decreased when cultured with conditioned medium. (**A**) MV4;11 cells were treated with 3 μM cytarabine in the presence or absence of multicell coculture with stromal, MSC, and endothelial cells in a 6:3:1 ratio. (**B**) MV4;11 cells were cultured in media that was collected from adherent cells growing for 48 h and diluted 50% with complete media. Cells were treated with 3 μM cytarabine where indicated. (**C**) MV4;11 cells were treated with 3 μM cytarabine and cultured in non-coated or MaxGel-coated wells to determine cell viability. Error bars denote standard deviation from the mean of three independent experiments in duplicates. Each black dot represents individual value. * *p* < 0.05, *** *p* < 0.001, **** *p* < 0.0001 indicates statistical significance.

We next wanted to identify if these chemoprotective effects on pediatric AML cells would be enhanced in multicell coculture with bone marrow stromal cells, endothelial cells, and MSCs to mimic the bone marrow microenvironment. IMDM media supplemented with cytokines (described in Section 2) was found to be appropriate for coculture of MV4;11 with endothelial, stromal, and MSCs and retained high cell viability (>83% for each cell type) (Figure S1A,B). While there was a 42.6% reduction in MV4;11 cell viability after 1.5 μM cytarabine treatment, there was only a 1.3% decrease in viability in multi cell coculture (Figure 1C, *p* < 0.01). The chemoprotection index in multicell coculture was 41.3, which was higher than that of individual cell types (Figure 1D). The difference in chemoprotection between multiple groups was statistically significant by two-way ANOVA (Figure S3). We also evaluated if such chemoprotection was present when the cytarabine concentration was increased to 3 μM. The chemoprotective index in multicell coculture with 3 μM cytarabine was 38.4 (Figure 2A, compare bars 2 and 4, *p* < 0.0001). Similar to cytarabine, multicell coculture also improved the cell viability of daunorubicin-treated MV4;11 cells, with a chemoprotection index of 27.1. While there was a 38.0% reduction in MV4;11 cell viability after 5 nM daunorubicin treatment, there was only a 10.6% decrease in viability in multi cell coculture (Figure S2, compare bars 2 and 4, *p* < 0.001).

### 3.2. The Chemoprotective Effects of Multicell Coculture on AML Cells Are Dependent on Cell–Cell and Cell–ECM Contact

Bone marrow microenvironment-induced chemoprotection can be modulated by soluble factors, interaction of cell adhesion molecules through direct cell–cell contact, and through interactions with the ECM [2,3,19,20]. We wanted to investigate if chemoprotective effects mediated by endothelial, stromal, and MSCs are dependent on soluble factors. To achieve this, MV4;11 cells were cultured in monoculture with 50% conditioned media from endothelial, stromal, and MSC cultures. The chemoprotective index was –10.9 with conditioned medium, suggesting that conditioned medium failed to replicate chemoprotective effects observed in multi cell coculture (Figure 2B, compare bars 2 and 4, *p* < 0.05).

We next investigated if leukemic cell and ECM contact could induce chemoprotection, by coating wells with MaxGel containing ECM components such as fibronectin, elastin, proteoglycans, collagen, tenascin, glycosaminoglycans, and TGF-ß found in the bone marrow microenvironment. MV4;11 cells were cultured in MaxGel-coated wells and treated with cytarabine for 48 h. MV4;11 cells cultured in MaxGel-coated wells showed a chemoprotection index of 21.2 (Figure 2C, compare bars 2 and 4, *p* < 0.001), indicating cell–ECM contact may play a role in chemoprotection. Taken together, these results suggest that chemoprotection is dependent on direct cell–cell contact and partially dependent on cell–ECM contact.

### 3.3. Azacitidine and Panobinostat Synergistically Sensitize Leukemic Cells to Cytarabine in Multicell Coculture

**Figure 3:**
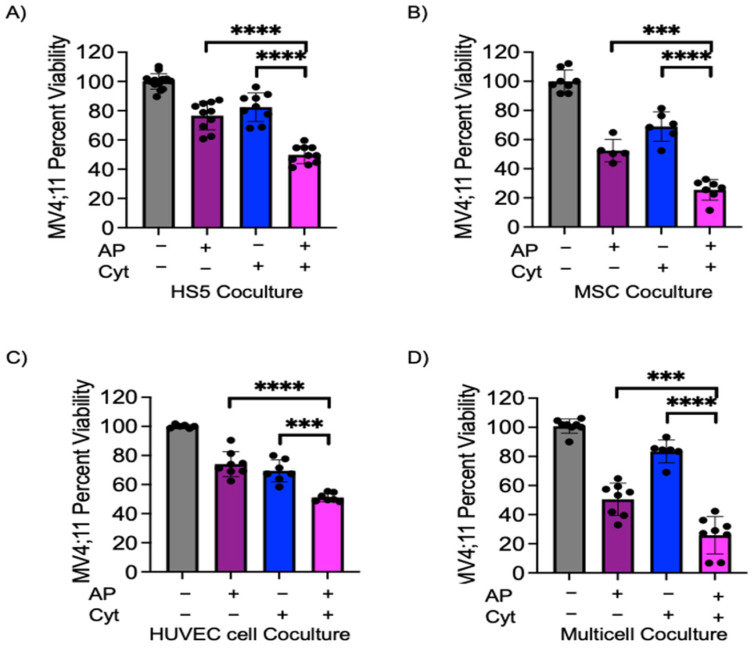
Azacitidine–panobinostat combination can sensitize MV4;11 cells to cytarabine in coculture model. MV4;11 cells were pretreated for 48 h with 1 μM azacitidine and 1 nM panobinostat. Leukemic cells were transferred onto adherent cells and treated with 1.5 μM cytarabine. (**A**) HS5, (**B**) MSCs, (**C**) endothelial cells, (**D**) adherent multicell layer of HS5, MSC and endothelial cells in a 3:1:6 ratio. Error bars denote SD of the Mean from 3–4 independent experiments in triplicates. Each black dot represents individual value. *** *p* < 0.001, **** *p* < 0.0001.

We have previously shown that azacitidine and panobinostat in combination initiates remission in murine models harboring MV4;11 xenografts [19,20]. We next wanted to identify if epigenetic modifier combination of azacitidine and panobinostat could sensitize AML cells and overcome bone marrow microenvironment-initiated chemoprotection. MV4;11 cells were pre-treated with azacitidine–panobinostat combination for 48 h and then treated with cytarabine for another 48 h in coculture with endothelial, stromal, and/or MSCs. In comparison to cytarabine treatment alone, pre-treatment with azacitidine–panobinostat followed by cytarabine treatment reduced viability by 32.6% in coculture with HS5, (Figure 3A, compare bars 3 and 4, *p* < 0.01), by 43.4% in coculture with MSCs (Figure 3B, compare bars 3 and 4, *p* < 0.01), by 18.2% in coculture with endothelial cells (Figure 3C, compare bars 3 and 4, *p* < 0.01), and by 57.6% in multicell coculture (Figure 3D, compare bars 3 and 4, *p* < 0.01). The differences in the degree of sensitization among different groups were statistically significant (Figure S5). Additionally, pre-treatment with azacitidine–panobinostat also reversed multicell coculture-mediated protection from daunorubicin treatment (Figure S4, *p* < 0.01).

We next wanted to identify if azacitidine–panobinostat pre-treatment synergized with cytarabine in inducing leukemic cell death. We calculated synergy with a relative risk ratio (RRR), as described previously [19]. A RRR value of less than 1.00 signifies a synergistic effect. MV4;11 had a RRR of 0.789, 0.707, 0.996, and 0.612 for HS5, MSC, endothelial cells, and multicell coculture, respectively, indicating the presence of strong synergy in individual and multicell coculture conditions. Taken together, these data demonstrate that azacitidine–panobinostat pretreatment sensitizes MV4;11 cells to cytarabine and daunorubicin treatment, even in multicell coculture conditions.

### 3.4. Azacitidine–Panobinostat Treatment Disrupts AML Cell Adhesion

**Figure 4:**
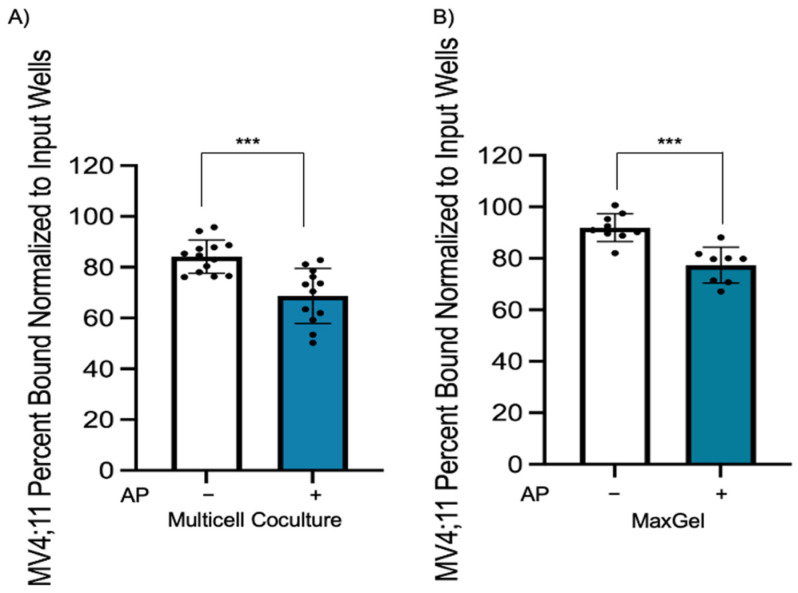
Epigenetic pretreatment causes a decrease in adhesion of MV4;11 cells to endothelial, stromal, and MSCs in multicell coculture and in MaxGel-coated wells. (**A**) MV4;11 cells were pretreated with 1 μM azacitidine and 1 nM panobinostat for 48 h, then stained and cultured for 4 h when plated onto stromal, MSCs, and endothelial cells in multicell coculture to determine if adhesion is impacted by epigenetic modifiers. The control is the percentage of bound untreated MV4;11 cells with respect to the input. (**B**) MV4;11 cells were plated in MaxGel-coated wells diluted in 1:100 DMEM media. Treatment adhesion was normalized and compared to input wells without PBS wash. Error bars display the SD from the mean of three independent experiments in duplicate. Each black dot represents individual value. *** *p* < 0.001.

Microenvironment-mediated chemoprotection is modulated by interaction of cell adhesion molecules through direct cell–cell contact and by ECM components. We wanted to determine if epigenetic drugs can overcome chemoprotection initiated through cell–cell or cell–ECM interactions by disrupting interactions between leukemic cell and bone marrow components. For this purpose, MV4;11 cells were pre-treated with azacitidine–panobinostat for 48 h and plated in multicell coculture or MaxGel. After 4 h, the unbound cells were carefully removed, and the number of adherent leukemic cells were quantified by flow cytometry. We found a significant reduction in the percentage of bound azacitidine–panobinostat-treated AML cells compared to untreated cells in both multicell coculture (Figure 4A, *p* < 0.001) and in MaxGel-coated dishes (Figure 4B, *p* < 0.001). These data suggest that azacitidine–panobinostat reduced leukemic cell binding to bone marrow components.

### 3.5. Epigenetic Drug Treatment Synergistically Sensitizes a Variety of KMT2A Rearranged AML Cells Ex Vivo

**Figure 5:**
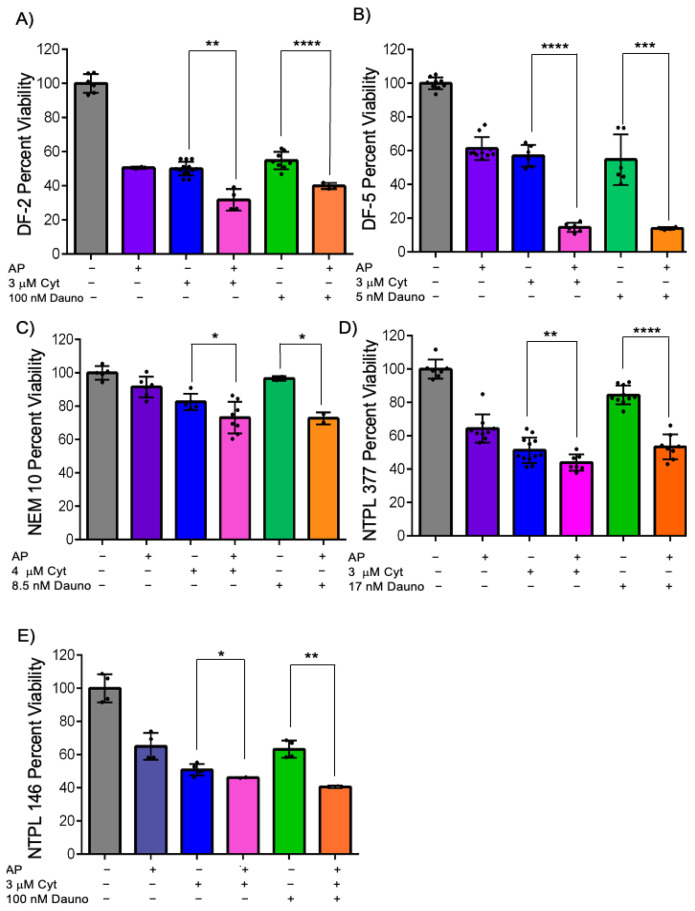
Azacitidine and panobinostat can synergistically sensitize *KMT2A*-rearranged PDX lines DF-2 (**A**), DF-5 (**B**), NEM10 (**C**), NTPL-377 (**D**), NTPL-146 (**E**) to cytarabine and daunorubicin. *KMT2A*-rearranged AML PDX lines were either left untreated or pretreated with 2.5 μM azacitidine (aza) and 2.5 nM panobinostat (pano) for DF-2 and NTPL-146, 5 μM aza, and 5 nM pano for DF-5, 0.5 μM aza, and 0.5 nM pano for NEM-10, 2.5 μM aza, and 1 nM pano for NTPL-377 for 24 h. Cells were then transferred onto endothelial, stromal, and MSCs and treated with 3 μM cytarabine for 48 h, except for NEM-10 which was 4 μM. Cells were treated with 100 nM daunorubicin (8.5 nM for NEM-10, 17 nM for NTPL-377 for 48 h. Error bars display the SD from the mean of two independent experiments in triplicate. Each black dot represents individual value. * *p* < 0.05, ** *p* < 0.01, *** *p* < 0.001, **** *p* < 0.0001.

Based on the synergistic sensitization of azacitidine–panobinostat to cytarabine or daunorubicin observed in MV4;11 cells in multicell coculture, we wanted to determine if this could be replicated using PDX lines. We have previously shown that treatment with 4 cycles (20 doses) of the azacitidine–panobinostat combination was curative in MV4;11 engrafted mice, whereas mice transplanted with a non-*KMT2A*-rearranged AML cell line did not achieve complete remission and the percentage of leukemic blasts in peripheral blood increased over time [16]. Therefore, we analyzed the chemo-sensitization of azacitidine–panobinostat ex vivo in five pediatric *KMT2A* rearranged AML PDX lines (Figure S6) by pretreating them with azacitidine–panobinostat in monoculture for 24 h, and then adding cytarabine or daunorubicin in multicell coculture with a monolayer of endothelial, stromal, and MSCs for 48 h. We found that all five PDX lines had significantly increased sensitivity to cytarabine following pretreatment with azacitidine–panobinostat (Figure 5A–E, compare bars 3 and 4, *p* < 0.05). Similarly, we found that these samples showed significantly increased sensitization percentage with daunorubicin, after pretreatment with azacitidine–panobinostat (Figure 5A–E, compare bars 5 and 6, *p* < 0.05). The differences in the degree of sensitization among various PDX lines were statistically significant by two-way ANOVA (Figures S7 and S8). These results suggest that azacitidine–panobinostat can synergistically sensitize *KMT2A*-rearranged AML cells harboring different fusion partners to cytarabine or daunorubicin ex vivo.

### 3.6. Azacitidine–Panobinostat Combination Mobilizes Leukemia Cells to the Peripheral Blood and Chemosensitizes MV4;11 and NTPL-146 Xenografted Mice

**Figure 6:**
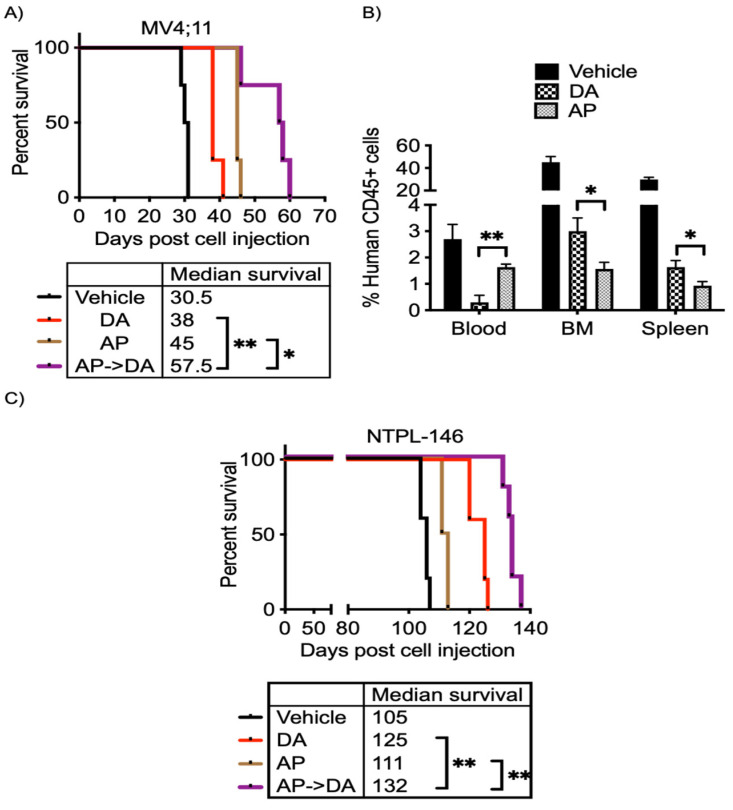
Pretreatment with azacitidine panobinostat followed by cytarabine and daunorubicin improves survival in vivo. (**A**) Kaplan–Meier survival plot shows the percentage of mice surviving following indicted treatments. Treatment started at 25 days. (**B**) The graph shows the percentage of human CD45+ leukemic cells in mouse blood, bone marrow, and spleen after treatment with epigenetic drugs and chemotherapy. (**C**) Kaplan–Meier plot shows the percent survival of mice post treatment. Treatment began at 26 days post cell injection. * *p* < 0.05, ** *p* < 0.01.

The effect of epigenetic therapy (azacitidine–panobinostat combination) on enhancing the efficacy of chemotherapy (cytarabine–daunorubicin combination) in vivo was tested in mice engrafted with MV4;11 and NTPL-146. Engraftment of human cells and disease progression was monitored by periodic determination of the percentage of human leukemic cells versus mouse cells in peripheral blood by flow cytometry using species-specific CD45 antibodies. The mice were randomly separated into four treatment groups (1) untreated, (2) epigenetic therapy (AP; azacitidine, panobinostat 2.5 mg/Kg each, daily for 5 days), (3) chemotherapy (DA; daunorubicin 1.5 mg/Kg daily for 3 days, cytarabine 50 mg/Kg daily for 5 days), and (4) epigenetic therapy followed by chemotherapy (AP- > DA). While toxicity due to epigenetic drug and chemotherapy treatments caused fatigue in mice, consistent loss in animal weight was not observed; mice regained weight once treatment was completed (Figure S9). While both chemotherapy and epigenetic therapy increased median survival by 7.5 and 14.5 days, respectively, the epigenetic therapy pretreatment before chemotherapy was more effective and resulted in 19-day prolonged survival (Figure 6A).

One day following conclusion of treatment, mice were euthanized to quantify leukemic blasts in peripheral blood, spleen, and bone marrow after epigenetic therapy or chemotherapy. Consistent with median survival, the percentage of AML cells in the bone marrow and spleen was lower following azacitidine–panobinostat treatment compared to chemotherapy alone (Figure 6B). Interestingly, the peripheral blood counts were higher in mice receiving azacitidine–panobinostat (Figure 6B). This suggests that epigenetic drug treatment disrupts leukemic cell interactions in the bone marrow microenvironment and increases mobility of leukemic blasts to the peripheral blood and decreases bone marrow load. Additionally, increased AML cells in the blood stream lead to greater chemosensitivity.

Similar to xenografts engrafted with MV4;11, chemosensitization with azacitidine–panobinostat increased median survival by 27 days in NTPL-146 PDX model compared to vehicle-treated mice (Figure 6C). Overall, AML xenografts receiving azacitidine and panobinostat followed by cytarabine and daunorubicin had a significant survival advantage compared to mice treated with epigenetic drugs or chemotherapy alone (Figure 6A,C; *p* < 0.05).

## 4. Discussion

Interactions within the bone marrow microenvironment mediate chemotherapy resistance in acute leukemias by promoting the survival of AML blasts [2,3,6,7,17,19,20,21,22,23,24,25,26,27]. Epigenetic drugs are a promising approach to sensitize leukemic blasts to chemotherapy because an aberrant epigenetic landscape is commonly observed in leukemogenesis [10,18,28,29,30,31,32]. Our results support previous studies illustrating that the DNA hypomethylating agent decitabine followed by cytarabine significantly decreases viability compared to cytarabine alone in vivo [32]. We showed previously that treatment of B-ALL cells with the epigenetic drug combination reversed chemoprotection in vitro and in vivo [19,33]. To our knowledge, this is the first report on the azacitidine and panobinostat combination sensitizing *KMT2A*-rearranged AML in multicell coculture to better mimic the bone marrow microenvironment and significantly increasing survival in vivo.

In contrast to bone marrow microenvironment-mediated chemoprotection, intrinsic resistance to cytarabine is common in AML cells [30,31]. Buelow et al. have shown DNA methylation within the promoter region of SLC22A4 mediates the expression of uptake transporter OCTN1 (SLC22A4) affecting cytarabine accumulation in AML cells [30]. Similarly, in *KMT2A*-rearranged ALL, decitabine causes DNA demethylation and increases sensitivity to cytarabine and daunorubicin [31]. Proapoptotic effects are observed following panobinostat treatment in monoculture [29]. Azacitidine and panobinostat treatment of AML cells in vitro results in reduced expression of *SUV39H1,* an epigenetic modifier *MYC,* and modifications in acetylated histone H3K9/27 [34]. In *KMT2A*-rearranged acute leukemia, sensitization following curaxin and panobinostat treatment is mediated by increasing histone acetylation, decreasing *MYC* expression, and not by altered expression of target genes *HOXA9* and *MEIS1* [10]. A recent CRISPR/Cas9 screen in B-ALL has identified a class III HDAC, *SIRT1,* overexpression in *KMT2A* rearrangements increases panobinostat sensitivity by mitochondrial activity initiation [35]. These chemoresistance mechanisms are different from the microenvironment-mediated chemoprotection observed in our multicell coculture study and epigenetic drugs are able to overcome them.

Our findings illustrating endothelial, stromal, and MSCs help AML cells evade chemotherapy are similar to recent coculture studies with histone deacetylase inhibitors. Treating osteoblasts with histone deacetylase inhibitors, vorinostat and panobinostat, alters the gene expression of osteoblasts and leads to chemosensitization of AML cells to cytarabine [18]. The interaction of T-ALL and endothelial cells results in transcriptomic changes modulating signaling pathways in leukemic and endothelial cells. Additionally, panobinostat increases survival in vivo and leads to reduced viability in T-ALL following direct contact with endothelial cells [36]. While VEGF-A/VEGFR-2 signaling aids in the formation of the endothelial niche in the bone marrow microenvironment, it does not contribute to cytarabine-initiated protection in vitro [20]. Quy et al. recently identified in AMKL that direct and sustained contact with endothelial cells is essential [5]. Consistent with these reports, our data show that chemoprotective effects of stromal, endothelial, and MSCs on AML cells require direct cell–cell or cell–ECM contact. A PI3K inhibitor increased the efficacy of panobinostat in coculture with MSCs derived from a healthy donor, indicating MSCs play a role in activating PI3K and decreasing the antileukemic effect of panobinostat in acute promyelocytic leukemia [28]. While MSCs derived from both healthy donor and AML patients provide chemoprotection to leukemic blasts, MSCs derived from AML were immunosuppressive [37]. A potential caveat in our multicell coculture model is that adherent cells from a single healthy donor were used. The effect of the AML patient-derived MSCs on AML cell survival may be distinct.

Cell adhesion molecules such as N-cadherin, VCAM-1, vascular endothelial (VE)-cadherin, E-selectin, and galectin-3 are known to play a role in leukemic cells adhering to stromal, endothelial, or MSCs to initiate chemoresistance [4,7,8,17,22,24,38]. A recent in vivo study identified *FERMT3* and *TLN1* as key regulators for integrin signaling in *KMT2A*-*MLLT3*-rearranged AML and β4galt1 [7]. Currently, work in the laboratory is ongoing to identify if the expression of CAMs that mediate AML cell interactions with bone marrow components is modulated by azacitidine and panobinostat. We observed that ECM showed chemoprotection, though not to the same extent as the cells. Previous reports have indicated that AML cell interactions with ECM components trigger survival signaling and prevent apoptosis following doxorubicin treatment by integrin-mediated cell adhesion [2]. Specifically, the protective effect of collagen in doxorubicin-treated AML cells in vitro is due to decreased DNA damage by inhibiting Rac1 signaling [39]. Other studies in solid tumors have shown that ECM may interfere with drug efficacy by entrapment of drugs [40]. The mechanism by which ECM mediates AML cell chemoprotection remains to be determined.

Our in vitro model with multicell coculture includes the three major cell types found within the bone marrow. However, other cell types such as adipocytes, osteoblasts, and immune cells which were not included may also be involved in bone marrow microenvironment-induced chemoprotection. Another potential caveat is that murine xenograft models are characterized by the human patient samples within the murine bone marrow microenvironment. Therefore, data from our in vitro and in vivo models may not be directly comparable. To further understand the effect of azacitidine–panobinostat on the bone marrow microenvironment additional in vivo studies investigating alterations in the microenvironment and interactions between leukemic cells and the bone marrow microenvironment following azacitidine–panobinostat treatment in murine models are necessary.

Azacitidine and panobinostat have a proapoptotic effect in AML and can overcome stromal-, endothelial-, and MSC-mediated chemoprotective effects in the bone marrow microenvironment. These chemosensitization effects can be replicated in pediatric AML PDX lines ex vivo as well as in vivo in mice engrafted with *KMT2A*-rearranged AML cell line or PDX line. Treatment with this epigenetic drug combination leads to reduced adhesion to the endothelial, stromal, and MSC layer and MaxGel, a bone marrow matrix mimetic, accompanied by an increase in the mobilization of leukemic blasts from the bone marrow to the peripheral blood. Mobilization of leukemic cells by agents such as plerixafor and GMI-1271 that disrupt cell adhesion mediated by CXCR4 and E-selectin, respectively, can sensitize AML cells to chemotherapy in vivo [8,41]. Similarly, disruption of annexin II/p11 interactions also caused ALL cell mobilization and chemosensitization [42,43]. Thus, it is possible that the epigenetic drugs play a similar role by antagonizing interactions of AML cells with the niche. Taken together, our results demonstrate that azacitidine–panobinostat synergistically overcomes microenvironment-initiated chemoprotective effects and is a candidate therapeutic approach for *KMT2A*-rearranged AML.

## 5. Conclusions

Epigenetic therapy synergistically overcomes bone marrow microenvironment-initiated chemoprotection in PDX samples of pediatric AML ex vivo and increases survival in murine models. Direct contact between leukemic cells and bone marrow components is required for chemoprotection. Epigenetic therapy reduces leukemic cell binding to bone marrow components and mobilizes leukemia cells from the bone marrow to the peripheral blood. Our study suggests this epigenetic drug combination could be incorporated in current treatment regimens for pediatric AML patients harboring *KMT2A* rearrangements.

## Data Availability

All data are available in this manuscript or the Supplementary Materials.

## Supplementary Materials

The following supporting information can be downloaded at: https://www.mdpi.com/article/10.3390/cancers15123112/s1, Figure S1: MV4;11, stromal, endothelial, and MSCs are viable when cultured in multicell coculture; Figure S2: MV4;11 cells in multicell coculture showed greater viability when exposed to daunorubicin; Figure S3: Two-way ANOVA analysis comparing the chemoprotection effect of HS5, MSC, HUVEC, and multicell coculture (row factor) on percentage of leukemic cell viability in comparison to monoculture (column factor); Figure S4: Azacitidine–panobinostat combination can sensitize MV4;11 cells to daunorubicin; Figure S5: Two-way ANOVA analysis comparing the sensitization effect of azacitidine–panobinostat synergy with cytarabine in HS5, MSC, HUVEC, and multicell coculture (row factor) on percentage of leukemic cell viability in comparison to cytarabine or azacitidine–panobinostat treatment alone (column factor); Figure S6: Patient characteristics, cytogenetics, and mutations of pediatric AML xenografts; Figure S7: Two-way ANOVA analysis comparing the sensitization effect of azacitidine–panobinostat synergy with cytarabine in multicell coculture (row factor) on percentage of PDX cell viability in comparison to cytarabine or azacitidine–panobinostat treatment alone (column factor); Figure S8: Two-way ANOVA analysis comparing the sensitization effect of azacitidine–panobinostat synergy with daunorubicin in multicell coculture (row factor) on percentage of PDX cell viability in comparison to daunorubicin or azacitidine–panobinostat treatment alone (column factor); Figure S9: Changes in animal weight following epigenetic drug and chemotherapy treatment in NTPL-146 engrafted mice (n = 5 each). Error bars indicate SD of the Mean.
