# Supplementary material for: Bone Marrow Microenvironment-Induced Chemoprotection in KMT2A Rearranged Pediatric AML Is Overcome by Azacitidine–Panobinostat Combination
Source: Cancers (Basel). 2023 Jun 8;15(12):3112. doi: 10.3390/cancers15123112 (PMC10296268; doi:10.3390/cancers15123112)

Supplemental Figure S1. MV4;11, stromal, endothelial and MSCs are viable when cultured in multicell coculture.
(A) Gating strategy to distinguish between four cell types. Cells were stained with CD31 and CD90 and expression was quantified with flow cytometer. (B) MV4;11, HS5, MSC and HUVEC cells were cultured in multicell coculture for 48 hours in IMDM media supplemented with cytokines and viability was analyzed with flow cytometer.

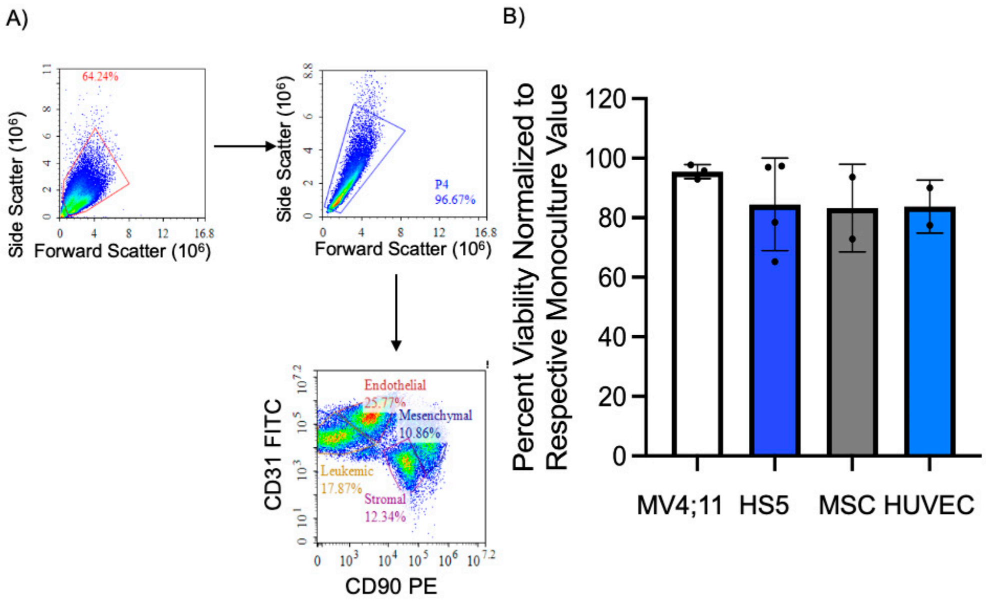

Supplemental Figure S2. MV4;11 cells in multicell coculture showed greater viability when exposed to daunorubicin. MV4;11 cells were treated with 5 nM daunorubicin (Dauno) in the presence or absence of multi cell coculture with, stromal, MSC and endothelial cells in a 6:3:1 ratio. Error bars display the SD from the mean of two independent experiments in duplicates. ***P<0.001, level of confidence to determine statistical significance.

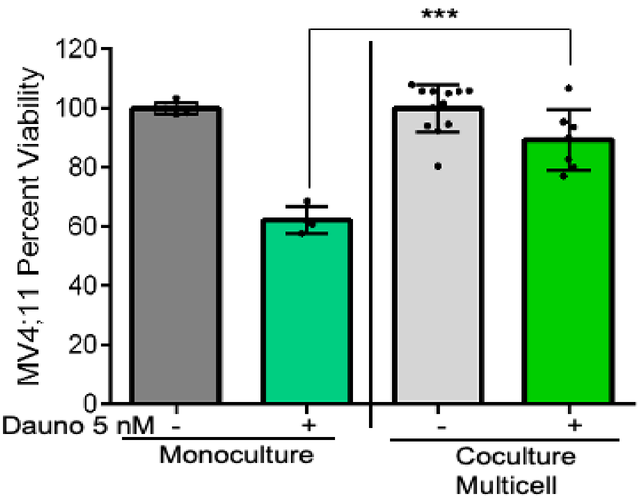

Supplemental Figure S3. Figure Two-way ANOVA analysis comparing the chemoprotection effect of HS5, MSC, HUVEC and multicell coculture (row factor) on percentage of leukemic cell viability in comparison to monoculture (column factor) following exposure to cytarabine.

ANOVA table	SS (Type III)	DF	MS	F(DFn,DFd)	P value
Interaction	950.3	3	316.8	F(3, 52) =5.537	P = 0.0022
Row Factor	759.3	3	253.1	F (3, 52) = 4.424	P=0.0076
Column Factor	11902	1	11902	F (1,52) = 208.0	P<0.0001

Supplemental Figure S4. Azacitidine-panobinostat combination (AP) can sensitize MV4;11 cells to daunorubicin (Dauno). MV4;11 cells were pretreated for 48 hours with 1 µM azacitidine and 1 nM panobinostat. Leukemic cells were transferred onto adherent multi cell layer of HS5, MSCs and endothelial cells in a 3:1:6 ratio and treated with 5 nM daunorubicin. Error bars denote SD of the mean from replicate values. *P<0.05, **P<0.01, level of confidence to determine statistical significance.

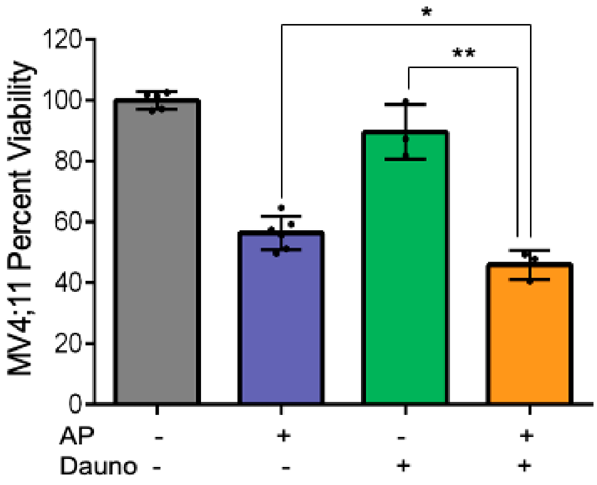

Supplemental Figure S5. Two-way ANOVA analysis comparing the sensitization effect of azacitidine-panobinostat synergy with cytarabine in HS5, MSC, HUVEC and multicell coculture (row factor) on percentage of leukemic cell viability in comparison to cytarabine or azacitidine-panobinostat treatment alone (column factor).

ANOVA table	SS (Type III)	DF	MS	F(DFn, DFd)	P value
Interaction	3689	6	614.9	F(6,79)=7.820	P<0.0001
Row Factor	6305	3	2102	F(3,79) =26.73	P<0.0001
Column Factor	22117	2	11059	F(2,79) =140.6	P<0.0001

Supplemental Figure S6. Patient characteristics, cytogenetics, and mutations of pediatric AML xenografts

Xenograft	Ethnicity	Age (years)/ Sex	AML subtype	Sample collected at	Fusion protein
NTPL-146	Caucasian	10/F	M5	Diagnosis	*KMT2A-MLLT1*
NTPL-377	Hispanic	1.5/F	M5	Diagnosis	*KMT2A-MLLT3*
DF-2 (CBAM-68552)	Caucasian	1/M	M5	Relapse following chemotherapy	*KMT2A-MLLT4*
DF-5 (CBAM-44728)	Unknown	15/F	M5	Second relapse following allogeneic HSCT	*KMT2A-MLLT10*
NEM10	Hispanic	7/F	M5	Diagnosis	*KMT2A-MLLT10*

Supplemental Figure S7. Two-way ANOVA analysis comparing the sensitization effect of azacitidine-panobinostat synergy with cytarabine in multi cell coculture (row factor) on percentage of PDX cell viability in comparison to cytarabine or azacitidine-panobinostat treatment alone (column factor).

ANOVA table	SS (Type III)	DF	MS	F(DFn, DFd)	P value
Interaction	3302	8	412.7	F(8,84) = 9.987	P<0.0001
Row Factor	17376	4	4344	F(4,84)=105.1	P<0.0001
Column Factor	6853	2	3427	F(2,84)=82.82	P<0.0001

Supplemental Figure S8. Two-way ANOVA analysis comparing the sensitization effect of azacitidine-panobinostat synergy with daunorubicin in multi cell coculture (row factor) on percentage of PDX cell viability in comparison to daunorubicin or azacitidine-panobinostat treatment alone (column factor).

ANOVA table	SS (Type III)	DF	MS	F(DFn, DFd)	P value
Interaction	3458	8	432.2	F(8,65)= 8.132	P<0.0001
Row Factor	13276	4	3319	F(4,65)= 62.44	P<0.0001
Column Factor	6803	2	3402	F(2,65) = 64.00	P<0.0001

Supplemental Figure S9. Changes in animal weight following epigenetic drug and chemotherapy treatment in NTPL-146 engrafted mice (n=5 each). Error bars indicate SD of the mean.

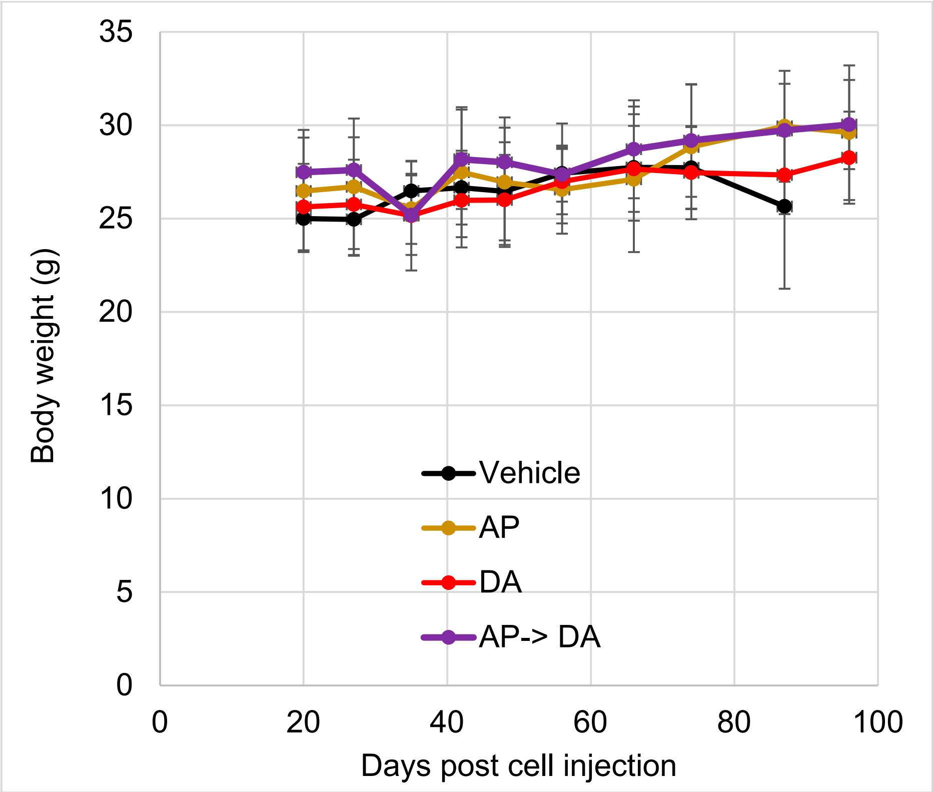

Supplement: Supplementary file 1 [file cancers-15-03112-s001.zip › cancers-2359091-supplementary.pdf]
